# Anti-Cancer Effects of 3, 3’-Diindolylmethane on Human Hepatocellular Carcinoma Cells Is Enhanced by Calcium Ionophore: The Role of Cytosolic Ca^2+^ and p38 MAPK

**DOI:** 10.3389/fphar.2019.01167

**Published:** 2019-10-09

**Authors:** Yuanyue Jiang, Yanfei Fang, Yang Ye, Xinming Xu, Bingfang Wang, Jie Gu, Michael Aschner, Jian Chen, Rongzhu Lu

**Affiliations:** ^1^Department of Preventive Medicine and Public Health Laboratory Science, School of Medicine, Jiangsu University, Zhenjiang, China; ^2^Department of Pathology, Kunshan Hospital of Traditional Chinese Medicine, Suzhou, China; ^3^Department of Gastroenterology, The First People's Hospital of Taicang City, Taicang Affiliated Hospital of Soochow University, Suzhou, China; ^4^Department of General Surgery, Affiliated Kunshan Hospital, Jiangsu University School of Medicine, Suzhou, China; ^5^Department of Digestive Disease, Affiliated Kunshan Hospital, Jiangsu University School of Medicine, Suzhou, China; ^6^Institute of Life Science, Jiangsu University, Zhenjiang, China; ^7^Department of Molecular Pharmacology, Albert Einstein College of Medicine, Bronx, NY, United States; ^8^Center for Experimental Research, Affiliated Kunshan Hospital, Jiangsu University School of Medicine, Suzhou, China

**Keywords:** 3,3′-diindolylmethane, cytosolic Ca^2+^, p38 MAPK, hepatocellular carcinoma cells, apoptosis, proliferation

## Abstract

**Purpose:** 3,3′-Diindolylmethane (DIM), derived from indole-3-carbinol (I3C) in the Brassica species of cruciferous vegetables, has anticancer effects, but its exact underlying mechanism of action is unknown. We explored the roles of cytosolic free calcium ([Ca^2+^]_i_) and p38 MAPK in the anti-cancer effects of DIM in human hepatocellular carcinoma cells.

**Methods:** Cell proliferation was measured with a Cell Counting Kit-8 (CCK-8) and the clonogenic formation assay. Cell apoptosis was examined by flow cytometric analysis and Hoechst dye staining. Cleaved-caspase3, cleaved-PARP, Bax, total, and phosphorylated p38 MAPK were assayed by western blotting. [Ca^2+^]_i_ was measured with Fluo-3/AM by fluorescence microscopy. A23187, a calcium ionophore, was used to increase [Ca^2+^]_i_ levels.

**Results:** DIM inhibited cell proliferation in both SMMC-7721 and HepG2 cells in a concentration- and time-dependent manner. DIM also enhanced phosphorylation of p38 MAPK (p-p38), which was attenuated by SB203580. The proliferation inhibition and apoptosis induction by DIM were also blunted. In addition, DIM increased [Ca^2+^]_i_ in HCC cells, and this effect was inhibited by the calcium chelator, BAPTA-AM, resulting in reduced p-p38 MAPK activation and apoptosis in DIM-treated cells, though the proliferation inhibition by DIM was unchanged. However, the DIM-induced cell proliferation inhibition and apoptosis were significantly enhanced by A23187, a selective calcium ionophore, which was attributed to exaggerated p-p38 MAPK.

**Conclusions:** The calcium ionophore enhanced DIM-induced anti-cancer effects in hepatocellular carcinoma cells, secondary to [Ca^2+^]_i_-dependent activation of p38 MAPK. Treatment with a combination of DIM and calcium ionophore may offer a new approach to enhance the chemotherapeutic efficacy in liver cancer.

## Introduction

Human hepatocellular carcinoma (HCC) is the sixth most common malignancy in the world and the third most common prevalent cause of cancer-related death worldwide (Forner et al., 2018). As HCC is highly resistant to standard chemotherapy, surgical resection or other treatments, most current therapies have limited efficacy. The identification of safe and effective treatments for advanced HCC remains challenging. Chemo-preventive agents with low toxicity and high efficiency in inhibiting tumor growth are promising candidates for cancer therapy (Sun and Hai, 2006).

3,3’-diindolylmethane (DIM), derived from Brassica species of cruciferous vegetables (broccoli, cabbage, and cauliflower) has displayed antitumor activity in several human cancers, including colon (Kim et al., 2007), pancreatic (Abdelrahim et al., 2006), and prostate cancer (Beaver et al., 2012). High intake of cruciferous vegetables has been associated with lower incidence of lung and colorectal cancer (Higdon et al., 2007). Pharmacologically, indole-3-carbinol inhibits WWP1, leading to the activation of the tumor suppressor PTEN, and in turn, attenuating PI3K-AKT-mTOR activation (Lee et al., 2019). We have also previously demonstrated that DIM inhibited the proliferation of gastric cancer cells by a miR-30e-ATG5-mediated autophagy (Ye et al., 2016). Thus, DIM may offer potential prevention and therapy in HCC.

Mitogen-activated protein kinases (MAPKs) are serine–threonine protein kinases that are involved in the regulation of cell proliferation, apoptosis (Davies et al., 2000), and other critical processes. The extracellular signal-regulated kinase (ERK), c-Jun NH (2)-terminal kinases (JNKs), p38 MAPK, are sub-families of MAPK (Kyriakis and Avruch, 2012). JNK and p38 MAPK are activated by cytokines and subsequently induce apoptosis (Davies et al., 2000). However, the role of p38 MAPK in mediating the protective cellular effects of DIM in cancers, including HCC, has not been determined.

Calcium ions act as second messengers in cytosolic signaling. The concentration of cytosolic Ca^2+^([Ca^2^+]i) is a key regulator of important cellular processes, such as cell proliferation and apoptosis (Zaichick et al., 2016; Tajbakhsh et al., 2018). In MG63 osteosarcoma cells, anandamide induces [Ca^2+^]_i_ elevation leading to p38 MAPK phosphorylation and subsequent apoptosis (Hsu et al., 2007). Aconitine significantly aggravates [Ca^2+^]_i_ overload and promotes apoptosis by phosphorylation of p38 MAPK (Sun et al., 2014). Up-regulation of [Ca^2+^]_i_ can induce a series of cellular responses, leading to cell death and apoptosis.

In the present study, we assessed the anticancer properties of DIM in two HCC cell lines, SMMC-7721 and HepG2 cells, and the potential involvement of the MAPK signaling pathway and [Ca^2+^]_i_. Our results identified a new mode of action for DIM, which could be beneficially exploited in future studies for therapeutic development.

## Materials and Methods

### Chemicals and Reagents

Dulbecco’s modified Eagle medium (DMEM) was purchased from Hyclone (MD, USA), primary antibodies anti-phosphory-p38 (1:1000), anti-cleaved caspase3 (1:1000), anti-cleaved PARP (1:1000), anti-Bax (1:1000), anti-p38 (1:1000), anti-caspase 3 (1:1000) were purchased from Cell Signaling Technology (MA, USA). Primary antibodies for PCNA and crystal violet dye were purchased from Bioss Biotechnology (Beijing, China). Primary antibody for β-actin and horseradish peroxidase (HRP)-labeled secondary antibodies were purchased from Santa Cruz Biotechnology (Santa Cruz, CA, USA). The flow cytometry reagent was purchased from BD Bioscience (San Jose, CA, USA). The p38 MAPK inhibitor SB203580, DIM (catalog no. BML-GR207), BAPTA-AM, A23187, Fluo-3/AM and Hoechst 33342 dye, were purchased from Sigma-Aldrich (St. Louis, MO, USA). SB203580 and DIM were dissolved in dimethyl sulfoxide (DMSO) as stock solution, respectively. The final concentration of DMSO did not exceed 0.1%.

### Cell Culture and Treatment

Two human HCC cell lines (SMMC-7721 and HepG2) were purchased from Shanghai Institute of Cell Bank (Shanghai, China). Both cell lines express alpha fetoprotein (AFP) protein (Liu et al., 2018; Qin et al., 2019), an established marker of liver cancer (Dezso et al., 2019; Takahashi et al., 2019), though notably SMMC-7721 is a HeLa derived cell line. The cells were grown as adherent monolayers in DMEM supplemented with 10% FBS (Thermo Fisher Scientific, WI, USA), 100 U/mL penicillin, and 100 μg/mL streptomycin. The cells were maintained at 37°C in an atmosphere of 5% CO_2_ and 95% humidity.

### Measurement of Cell Proliferation

Cell viability was assessed with the CCK-8 assay. The CCK-8 (Cell Counting Kit-8) was purchased from Dojindo (Kumamoto, Japan). Briefly, cells were seeded into 96-well plates at 5 × 10^3^ cells per well and cultivated for 24 h to adhere. Cells were then incubated with various concentration of DIM (0, 20, 40, 60, 80, 100 and 120µM) for 24, 48, or 72 h; or pretreated with SB203580 (5µM or 10µM) for 3 h, BAPTA-AM (10µM), A23187 (1µM) for 0.5 h, then DIM was added for 24 h. The media was removed, and the mixture of CCK-8 and DMEM (1:10, v/v) was added to each well. Plates were incubated for an additional 40 mins at 37°C; and the absorbance was measured at 450 nm using an Automated Microplated Reader (Bio-Tek ELx800uv, Bio-Tek Instrument Inc., Winooski, VT, USA) at wave length of 450 nm.

### Clonogenic Formation Assays

SMMC-7721 and HepG2 cells were seeded at 4×10^5^ cells/well into 6-well plates and incubated overnight. Cells were cultured in the presence or absence of DIM or SB203580 for 3 h and incubated with DIM for an additional 24 h. Cells were trypsinized, counted, and replated in triplicate in predetermined cell numbers to yield 300 colonies per well. Plates were incubated for 10–15 days to allow clonogenic growth. Colonies were washed X3 with cold PBS and fixed in methanol for 20 min. After washing with PBS, the colonies were counted after staining with 0.3% crystal violet solution at RT for 30 min.

### Measurement of Apoptosis

Apoptosis in hepatoma cells was assayed with Hoechst 33342 staining. Hepatoma cells (3000 cells/well) were plated in triplicate in 24-well plates and incubated overnight. Cells were incubated in the presence or absence of SB203580 for 3 h, and then DIM was added for an additional 24 h. After washing with PBS, the cells were incubated with Hoechst dye in the dark at RT for 15 min and imaged with an inverted fluorescence microscope (Olympus, Japan). Typical DNA fragmentation photographs were chose to indicate cell morphology, while apoptotic cells were counted with 1,000 cells at each group. The apoptotic index was calculated as follows: apoptotic index = apoptotic cell number/(apoptotic cell number + non apoptotic cell number). The similar data are presented with flow cytometry and the pictures from Hoechst are more directly. Apoptosis was evaluated with propidium iodide/annexin V-FITC apoptosis detection kit (BD Biosciences, CA, USA) according to the manufacturer’s instructions. Cells were seeded in 6-well plates and allowed to attach overnight. Cells were incubated in the presence or absence of SB203580 for 3 h, 10 μM BAPTA-AM, 1μM A23187 for 0.5 h, and subsequently DIM was added for an additional 24 h. The cells were collected and processed using the detection kit and analyzed with flow cytometry (FACScan, Becton Dickinson, USA) in the FACS facility at Jiangsu University. Apoptosis was quantified by flow cytometry. At least 20,000 events were counted for each sample.

### Western Blotting Analysis

Cells were washed with ice-cold 1 × PBS and lysed in radio immune precipitation assay buffer supplemented with phenylmethanesulfonyl and phosphatase inhibitor cocktail, and centrifuged at 12,000g for 15 min at 4°C. Protein concentration was determined by the bicinchoninic acid protein assay, and equal amounts of proteins were subjected to 12% SDS-PAGE, and then transferred onto nitrocellulose membranes. The membranes were blocked for nonspecific binding with 5% nonfat milk in Tris-buffered saline containing 0.05% Tween 20 for 2 h at RT and then probed with primary antibodies overnight at 4°C, followed by incubation with HRP-conjugated immunoglobulin G (IgG) for 1 h at RT. Chemiluminescence was detected with ECL reagents (Thermo Fisher Scientific, Etten-Leur, The Netherlands).

### Measurement of Cytosolic Ca^2+^ Levels

Cells were seeded at a density of 5×10^5^ cells/well in 6-well plates. The next day, cells were pretreated with BAPTA-AM (10µM) or A23187(1µM) for 0.5 h, then treated with DIM (60µM) for another 24 h, followed by removal of the medium, and three washes with PBS. Cells were loaded with 5 µM Fluo-3/AM for 1 h at 37°C in the dark, and then washed once with PBS to remove the extracellular Fluo-3/AM. Calcium imaging was acquired with a Nikon Eclipse TE2000-U inverted fluorescence microscope. Cells were treated as previously described, washed X2 with PBS, and incubated with Fluo-3/AM for 1 h, followed by trypsinization and loading with 1× binding buffer. At least 10,000 events were counted for each sample. The Ca^2+^-dependent mean fluorescence intensity was measured with Cytoflex (Beckman Coulter, CA, USA). The experiments were conducted independently three times.

### Statistical Analysis

All experimental data from three independent experiments were presented as mean ± SD. Prism 5.0 software (Graph Pad Software, San Diego, CA, USA) was used for data analysis. Comparisons among at least three groups were analyzed with one-way analysis of variance (ANOVA) followed by the Dunnett’s *post hoc* test or Student-Newman-Keuls post-hoc test depending on the test purpose. Statistical differences were considered significant when *P* < 0.05.

## Results

### Effects of DIM on Cell Proliferation in Liver Cancer Cells

**Figure 1:**
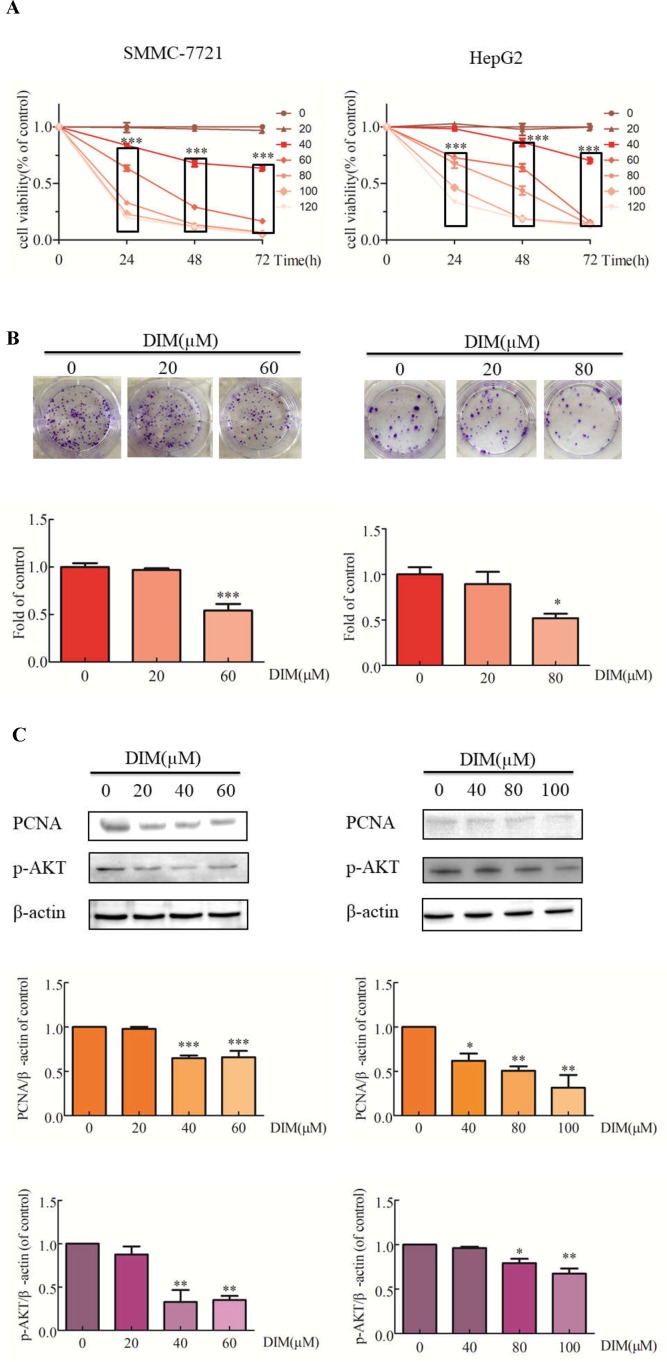
Effects of DIM on cell proliferation and related proteins in SMMC-7721 and HepG2 liver cancer cells. **(A)** Effects of DIM on cell proliferation were measured with the CCK-8 assay. Results are expressed as the percentage of blank control cells. **(B)** Colony formation assays in HCC cell lines treated with the indicated concentrations of DIM for 24 h. **(C)** Western blotting analysis of PCNA and p-AKT in HCC cells treated with the indicated concentrations of DIM for 24 h. β-actin was used as an internal control. Data represent mean ± SD of three independent experiments (*n* = 3). *P < 0.05, **P < 0.01 and ***P < 0.001 compared with the control group. DIM: 3,3’-diindolylmethane.

The effects of DIM on liver cancer cell growth were evaluated with the CCK-8 assay. DIM increased the cytotoxic effect compared with untreated controls (**Figure 1A**). Cell viability was significantly decreased in SMMC-7721 cells treated with 80μM DIM and by 25% in HepG2 cells treated with 60 μM DIM for 24 h. DIM significantly inhibited colony formation in SMMC-7721 cells (at 60 µM) by 46% and in HepG2 cells by 49% (80 µM) compared with controls (**Figure 1B**). The cytotoxicity of DIM was apparent at 24, 48, 72 h; however, since the protein lysates were difficult to acquire at 48 or 72 h, the 24-h timepoint was chosen for the following experiments. As shown in **Figure 1C**, western blotting analysis established that DIM significantly reduced the protein level of proliferation cell nuclear antigen (PCNA) and p-AKT in both cell lines.

### Effects of DIM on Cell Apoptosis and Related Protein Activity in Liver Cancer Cells

**Figure 2:**
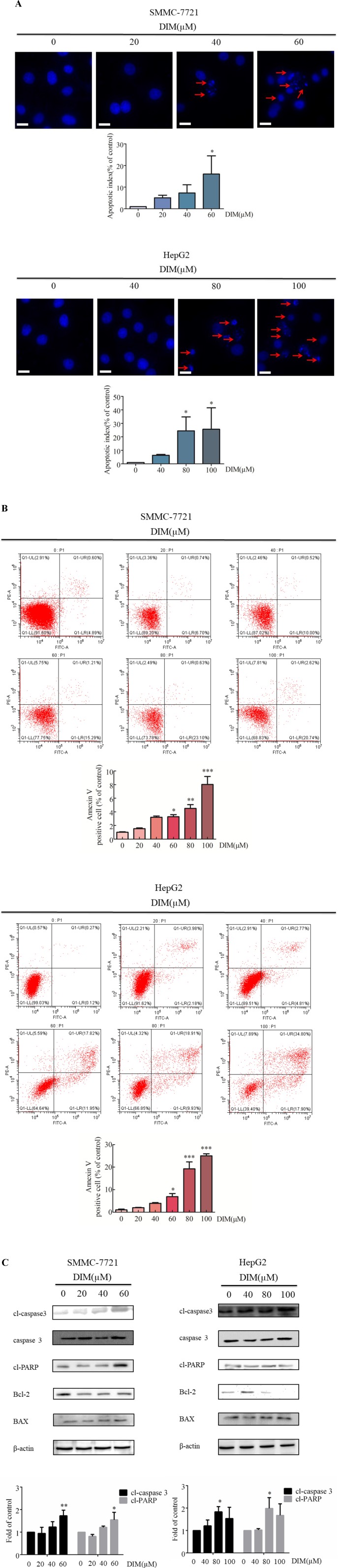
Effects of DIM on cell apoptosis and apoptosis-related protein levels in SMMC-7721 and HepG2 liver cancer cells. **(A)**. Effects of DIM on apoptosis assessed with Hoechst staining. Red arrows indicate apoptotic cells. Cells were divided and counted as apoptotic cells and non apoptotic cells in 1,000 events at each group. Apoptotic index = apoptotic cell number/(apoptotic cell number + non apoptotic cell number). **(B)** Effects of DIM on apoptosis assessed by flow cytometry analysis and Annexin V-FITC and PI staining. **(C)** Effects of DIM on apoptosis-related protein expressions. Western blotting was performed for the indicated proteins in HCC cells treated with various concentration of DIM for 24 h. β-actin was used as an internal control. Scale bar represents 15 µM. Data represent mean ± SD of three independent experiments (n = 3). *P < 0.05, **P < 0.01 and ***P < 0.001 compared with the control group.

The effects of various concentrations of DIM on apoptosis in HCC cells were examined by Hoechst staining. Upon 24 h treatment with 60 μM or 80 μM DIM of SMMC-7721 or HepG2 cells, respectively, the number of apoptotic cells with DNA fragmentation was significantly greater than in the control group (**Figure 2A**). To corroborate this observation, propidium iodide/Annexin V-FITC staining and flow cytometry in HCC cells treated with DIM were performed. As shown in **Figure 2B**, DIM significantly increased the apoptotic cell population up to 4–5-fold compared with control untreated cells.

To elucidate the apoptotic mechanisms associated with DIM, levels of apoptosis-related proteins were evaluated by western blotting. One of the key events in apoptosis is the activation of a cascade of intracellular cysteine proteases known as caspases (Jacobson et al., 1997). Upon proteolytic activation by upstream caspases, caspase-3 cleaves a variety of substrates, including PARP. As shown in **Figure 2C**, treatment with DIM increased cleaved-caspase3, as well as other apoptosis-associated protein, such as cleaved-PARP and Bax/Bcl2, in a concentration-dependent manner. These results establish that cleaved-caspase3 is activated in response to DIM, and that the caspase cascade signaling is responsible, at least in part, for DIM induced apoptosis.

### Effects of DIM on Phosphorylation of p38 MAPK in Liver Cancer Cells

**Figure 3:**
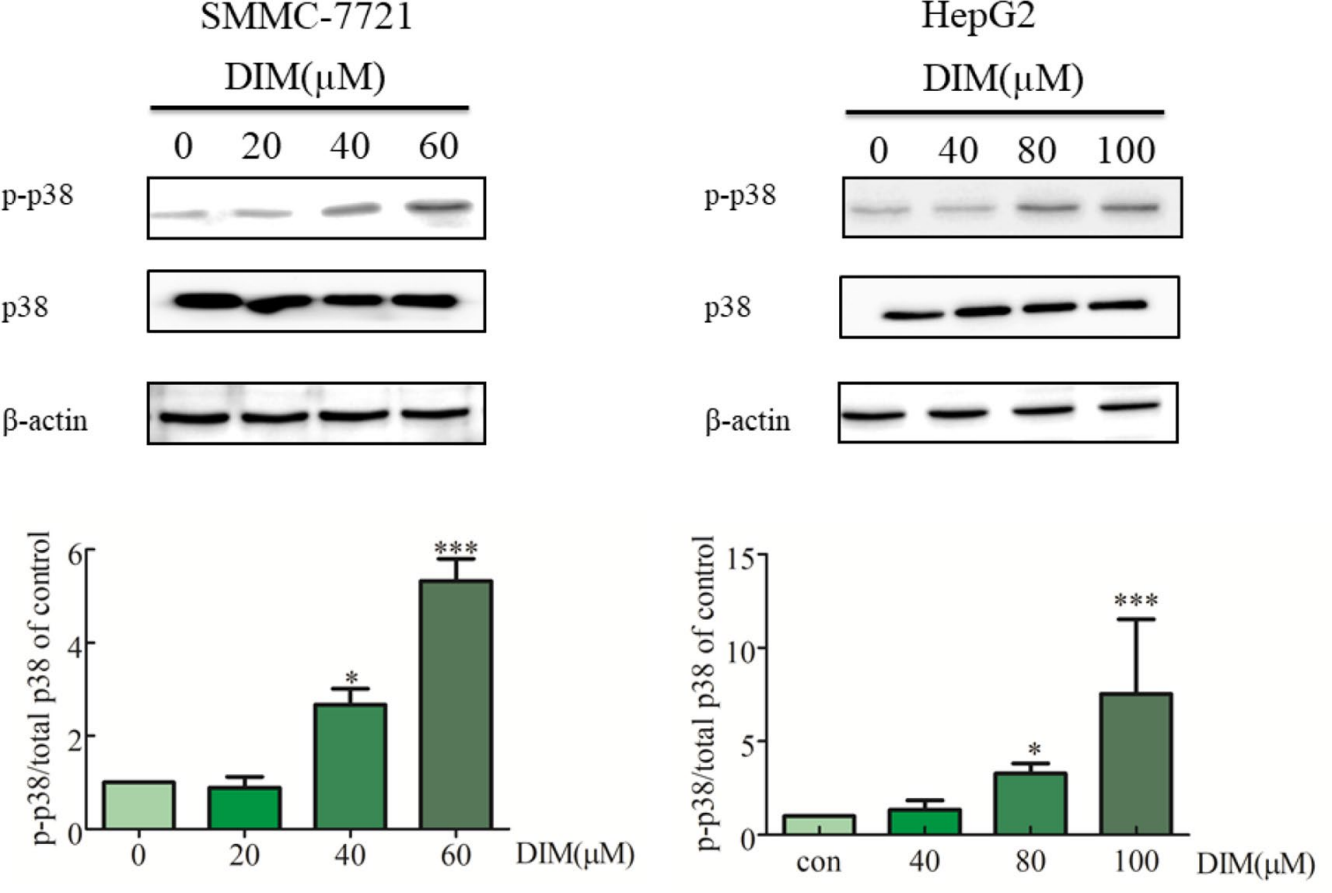
Effects of DIM on phosphorylation of p38 MAPK in hepatoma cancer cells. β-actin was used as an internal control. The level of phospho-p38 was normalized on the total p38.Data represent mean ± SD of three independent experiments (*n* = 3). *P < 0.05 and ***P < 0.001 compared with the control group.

To explore the potential mechanism underlying DIM-induced cell proliferation inhibition, the effects of DIM on MAPK activation were examined. HCC cells were treated with various concentrations of DIM for 24 h, and the levels of phospho-p38 MAPK and total p38 MAPK were evaluated by western blotting (**Figure 3**). DIM significantly increased the phosphorylation of p38 MAPK in both SMMC-7721 and HepG2 cell lines in a concentration-dependent manner compared with untreated cells, with no effect on total p38 MAPK level. The level of phospho-p38 was normalized to the total p38.

### Effects of p38 MAPK Inhibitor on DIM-Induced Phosphorylation of p38 MAPK and Apoptosis in Liver Cancer Cells

**Figure 4:**
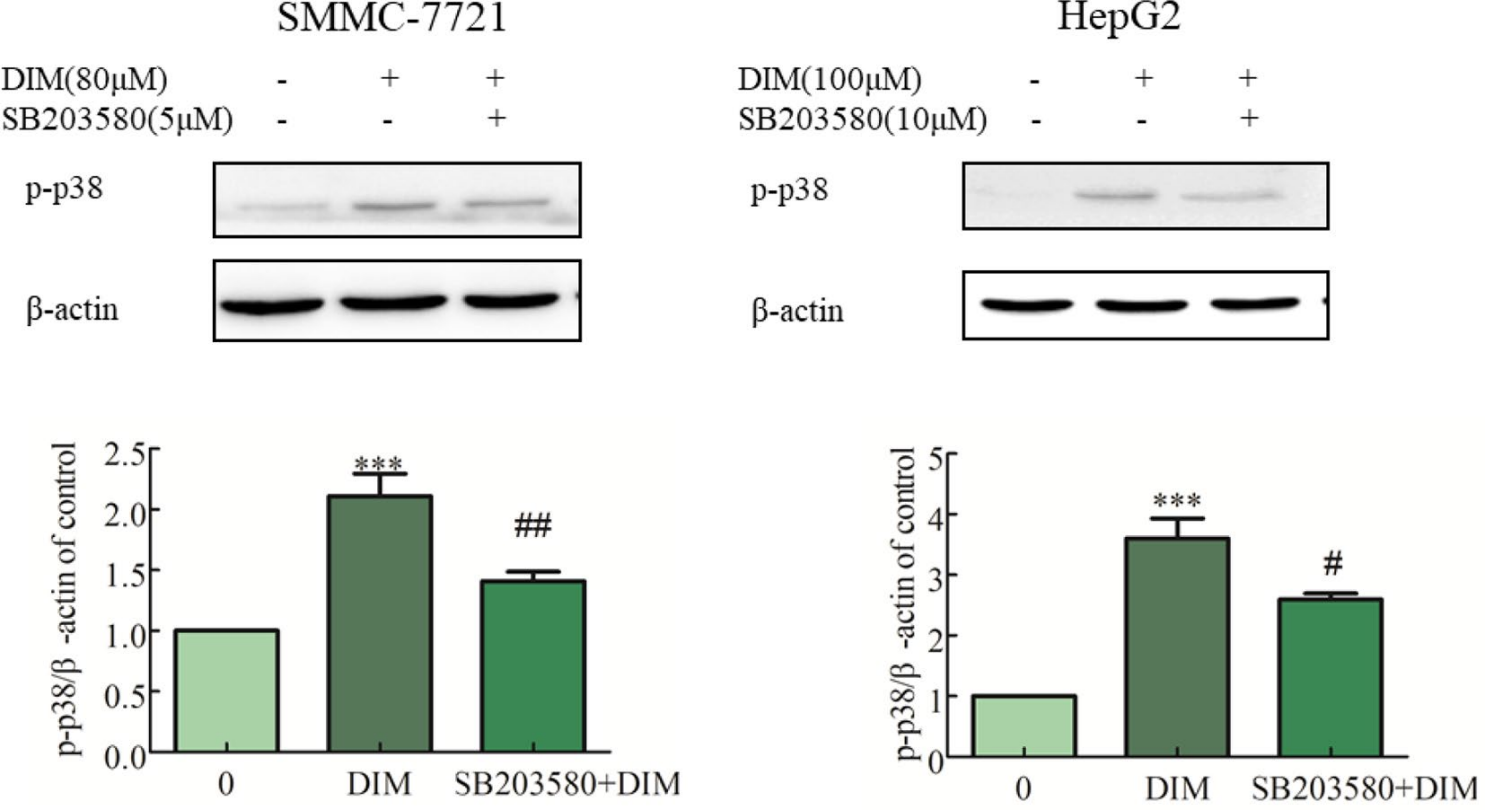
Effects of p38 MAPK inhibitor (SB203580) on DIM-induced phospho-p38 levels in hepatoma cancer cells. SB203580 was used 3 h prior to the addition of DIM for 24 h. β-actin was used as an internal control. Data represent mean ± SD of three independent experiments (*n* = 3). ***P < 0.001 compared with the control group; ^#^ *P* < 0.05, ^##^ *P* < 0.01 compared with DIM alone group.

**Figure 5:**
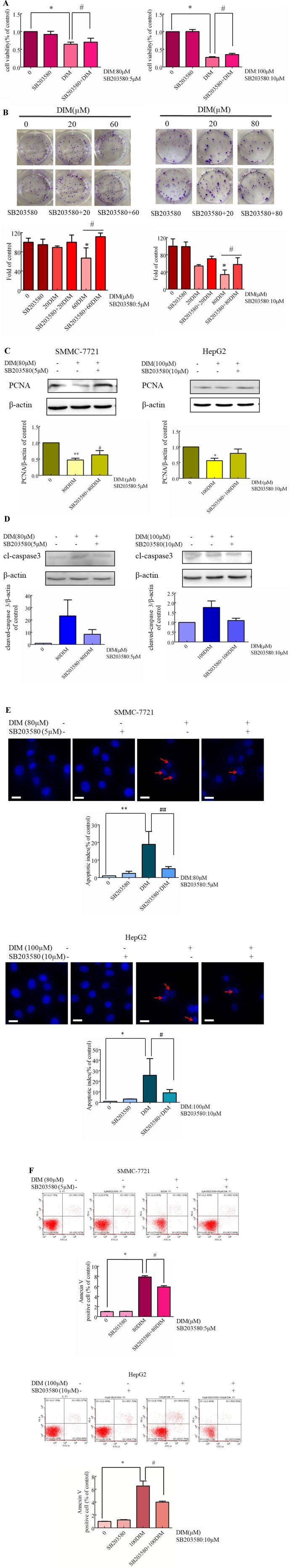
Effects of p38 MAPK inhibitor on DIM-induced apoptosis in hepatoma cancer cells. SB203580 was used 3 h prior to the addition of DIM for 24 h. **(A)** Effects of SB203580 on DIM-induced proliferation inhibition assessed by CCK-8 assay. **(B)** Effects of SB203580 on DIM-induced cell proliferation inhibition assessed by clonogenic formation assay. **(C)** Effects of SB203580 on DIM-induced PCNA protein expression. **(D)** Effects of SB203580 on DIM-induced changes of cleaved caspase-3. β-actin was used as an internal control. **(E)** Effects of SB203580 on DIM-induced apoptosis determined by Hoechst staining. **(F)** Effects of SB203580 on DIM-induced apoptosis determined by Annexin V-FITC/PI staining and flow cytometry. Scale bar represents 15 µM. Data represent mean ± SD of three independent experiments (*n* = 3). *P < 0.05, **P < 0.01 compared with controls; ^#^ *P* < 0.05, ^##^ *P* < 0.01 compared with DIM alone group.

To evaluate the role of phospho-p38 in DIM-induced growth inhibition, a specific phospho-p38 MAPK inhibitor (SB203580) was used 3 h prior to the addition of DIM for 24 h. Western blot analysis confirmed that the inhibitor partially impaired the activation of phospho-p38 MAPK in response to DIM (**Figure 4**). Based on the results in **Figure 1**, 80 μM or 100 μM DIM in DIM in SMMC-7721 or HepG2, respectively, led to ∼50% loss in cell viability. Pretreatment with the phospho-p38 inhibitor for 3 h partially attenuated the cell growth inhibition induced by 80 or 100 μM DIM in CCK-8 assays as well as colony formation assays (**Figures 5A, B**) in the following 24 h. In previous experiments, 5 μM SB203580 or 10 μM SB203580 in SMMC-7721 or HepG2, repectively, led to no cytotoxicity. SB203580 was used 3 h prior to the addition of DIM for 24 h. Western blotting analysis was consistent with the cell viability data, showing restoration of PCNA levels in cells treated with the p38 inhibitor and DIM compared with DIM treatment alone (**Figure 5C**). These results suggest that DIM-induced inhibition of proliferation is dependent, at least in part, upon phospho-p38 activity and that the proliferation inhibition is highly correlated with the phospho-p38 MAPK pathway in both SMMC-77721 and HepG2 cells.

To explore the potential mechanism underlying DIM-induced apoptosis, the evaluation of the expression of cleaved-caspase 3 in HCC cells pretreated with p38 inhibitor was observed. The results indicated that DIM-induced cleavage of caspase 3 was dependent upon phospho-p38 MAPK activity (**Figure 5D**). The p38 inhibitor also reversed the apoptosis induced by DIM partially, as evidenced by both Hoechst dye staining and flow cytometry analysis (**Figures 5E, F**). These results suggest that DIM-induced apoptosis in HCC is likely mediated through the phospho-p38 MAPK pathway.

### Effects of DIM on Cytosolic Free Calcium in Liver Cancer Cells

**Figure 6:**
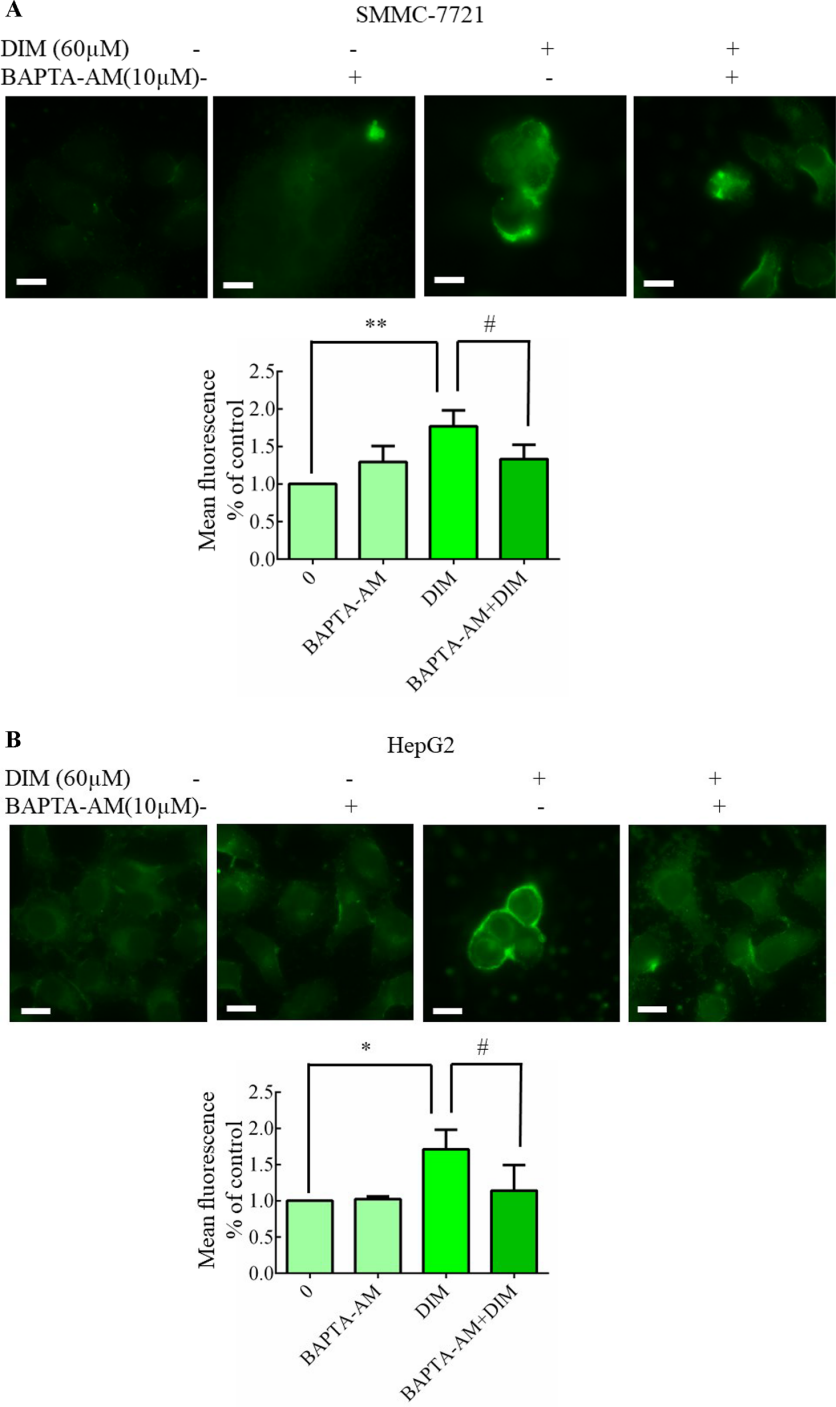
Effects of DIM on cytosolic free calcium in hepatoma cancer cells. **(A**, **B)** Effects of BAPTA/AM on DIM-induced changes of cytosolic Ca^2+^ levels. Similar results were observed in at least three independent experiments. BAPTA-AM: 1,2-bis (2-aminophenoxy)ethane-N, N, N’, N’-tetraacetic acid/acetoxymethyl ester. Scale bar represents 15 µM. Data represent mean ± SD of three independent experiments (*n* = 3). *P < 0.05, **P < 0.01 compared with controls; ^#^ *P* < 0.05 compared with DIM alone group.

To determine whether DIM affected [Ca^2+^]_i_ levels, calcium imaging was performed with Fluo-3/AM in calcium-containing medium using fluorescence microscopy. As shown in **Figures 6A, B**, 60 μM DIM increased [Ca^2+^]_i_ in both HCC cell lines. To further verify the role of cytosolic Ca^2+^ in this response, we chelated Ca^2+^ with 10 µM BAPTA-AM, in previous experiments, 10 μM BAPTA-AM showed no cytotoxicity in SMMC-7721 or HepG2 and found that BAPTA-AM strongly reduced the rise in free calcium concentrations.

### Effects of Chelating Cytosolic Ca^2+^ on DIM-Induced Cell Apoptosis in Liver Cancer Cells

**Figure 7:**
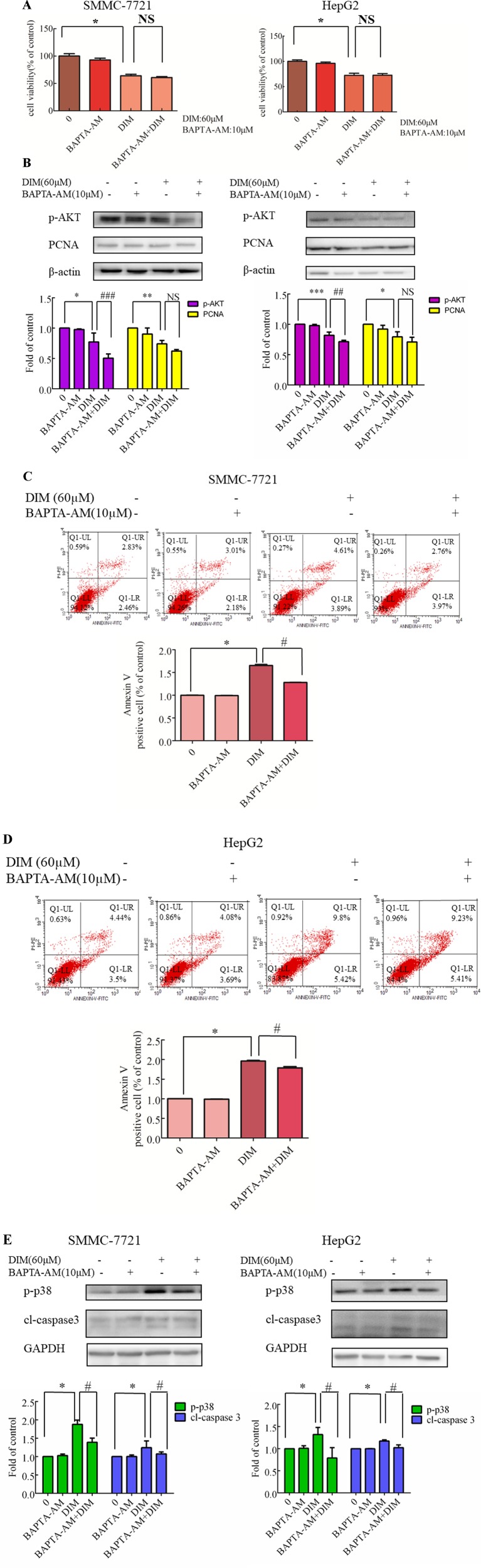
Effects of chelating Ca^2+^ with BAPTA-AM on DIM-induced changes of proliferation and apoptosis in HCC cells. BAPTA-AM (10 µM) was added 0.5 h prior to the addition of DIM (60 µM) for 24 h in the following experiments. **(A)** Effects of BAPTA-AM on DIM-induced cell proliferation measured with CCK-8 assays. Results are expressed as the percentage of blank control cells. **(B)** Effects of BAPTA-AM on DIM-induced changes of PCNA and p-AKT proteins. Similar results were observed in at least three independent experiments and β-actin served as a loading control. **(C**, **D)** Effects of BAPTA-AM on DIM-induced apoptosis detected by flow cytometry. Similar results were observed in at least three independent experiments. **(E)** Effects of BAPTA-AM on DIM-induced changes of p-p38 and cleaved-caspase 3 levels. GAPDH was used as an internal control. Data represent the mean ± SD of experiments conducted in triplicate (*n* = 3). *P < 0.05, **P < 0.01 and ***P < 0.001 compared with the control group; ^#^ *P* < 0.05, ^##^ *P* < 0.01, ^###^ *P* < 0.001 compared with DIM alone group.

To identify the source of the DIM-induced [Ca^2+^]_i_ increase, HCC cells were pretreated with BAPTA-AM (10 µM) for 0.5 h to prevent the increase in [Ca^2+^]_i_ transients. DIM (60 µM) was subsequently added for 24 h in the following experiments. No significant change in cell viability analysis was noted (**Figure 7A**). Western blotting analysis was consistent with the cell viability data. PCNA expression levels showed that BAPTA-AM loading did not alter the inhibition of proliferation (**Figure 7B**). In the presence of 60 µM DIM, BAPTA-AM loading did not prevent DIM-induced cytotoxicity. However, pretreatment with BAPTA-AM significantly suppressed DIM-induced apoptosis (**Figures 7C, D**). Western blotting showed that chelating Ca^2+^ with BAPTA-AM partially inhibited the DIM-induced phosphorylation of p38 MAPK (**Figure 7E**).

### Effects of Ca^2+^ Ionophore A23187 on DIM-Induced Cell Apoptosis and Phosphorylation of p38 MAPK in Liver Cancer Cells

**Figure 8:**
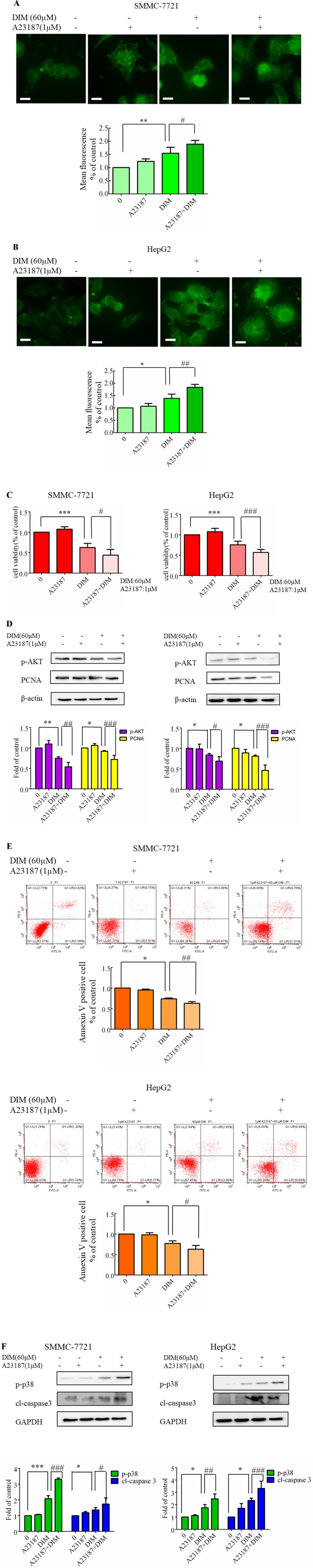
Effects of Ca^2+^ ionophore A23187 on DIM-induced cell apoptosis and phosphorylation of p38 MAPK in liver cancer cells. A23187 (1 µM) was added 0.5 h prior to the addition of DIM (60 µM) for 24 h in the following experiments. **(A**, **B)** Effects of A23187 on DIM-induced changes of [Ca^2+^]_i_. Analogous results were observed in a minimum of three independent experiments. **(C)** Effects of A23187 on DIM-induced changes of cell proliferation assessed by CCK-8 assays. Results are expressed as the percentage of blank control cells. **(D)** Effects of A23187 on DIM-induced changes of PCNA and p-AKT. β-actin served as a loading control. **(E)** Effects of A23187 on DIM-induced apoptosis detected by flow cytometry. Similar results were observed in a minimum of three independent experiments. **(F)** Effects of A23187 on DIM-induced apoptosis and phosphorylation of p38 MAPK. GAPDH was used as an internal control. Scale bar represents 15 µM. Data represent the mean ± SD of experiments conducted in triplicate (*n* = 3). *P < 0.05, **P < 0.01 and ***P < 0.001 compared with the control group, ^#^ *P* < 0.05, ^##^ *P* < 0.01, ^###^ *P* < 0.001 compared with the DIM alone group.

To further address the calcium elevation effects, we decided to focus our studies on the use of a calcium ionophore. In previous experiments, 1 μM A23187 showed no cytotoxicity in HCC cells. Therefore the cells were pretreated A23187 (1 µM) for 0.5 h to increase [Ca^2+^]_i_, and DIM (60 µM) was subsequently added for 24 h. The calcium ionophore (A23187, 1 µM) increased the 60 µM DIM-induced elevation in [Ca^2+^]_i_ transients (**Figures 8A, B**). The calcium ionophore also enhanced DIM-induced cytotoxicity by ∼20% (**Figure 8C**). Western blotting analysis was consistent with the cell viability data. As shown in **Figure 8D**, increased [Ca^2+^]_i_ led to a statistically significant decrease in AKT phosphorylation. A23187 significantly increased DIM-induced apoptosis (**Figure 8E**). Pretreatment with a calcium ionophore (**Figure 8F**) increased the phosphorylation of p38 MAPK in the presence of DIM by 75% in SMMC-7721 cells and 50% in HepG2 cells. In addition, cleaved-caspase3 increased by 50% in comparison with the DIM alone-induced protein level.

## Discussion

In the present study, we probed the signaling mechanisms underlying the DIM-induced proliferation inhibition and apoptosis induction in liver cancer cells. Our study demonstrated several novel findings. First, we showed that DIM inhibited cell proliferation and enhanced cell apoptosis in SMMC-7721 and HepG2 cell lines. Second, DIM-induced activation of apoptosis and proliferation inhibition were attenuated by inhibiting p38 MAPK with SB203580, indicating that p38 MAPK activation is involved, at least in part, in the cellular effects of DIM. In addition, DIM altered calcium homeostasis, enhancing cytosolic calcium levels, accompanied by up-regulation of phospho-p38 MAPK. Pretreatment with the calcium chelator BAPTA-AM and calcium ionophore A23187 corroborated these findings. Thus, these results demonstrate that the apoptotic effects of DIM are mediated by the Ca^2+^-dependent p38 MAPK signaling pathway.

Proliferation inhibition and induction of apoptosis are two key mechanisms by which chemotherapeutic agents induce cytotoxic effects in cancer cells (Li et al., 2005). The present study shows that DIM treatment induces proliferation inhibition and apoptosis in HCC cells, consistent with earlier reports on the anti-proliferative and pro-apoptotic abilities of DIM (Shorey et al., 2012; Zhu et al., 2012; Li et al., 2015). These results suggest that this diet-derived phytochemical might have clinical utility as a therapeutic or adjuvant therapeutic agent for HCC.

Apoptosis is a process of programmed cell death. Previous studies have shown that DIM induces apoptosis in several cancer cell lines (Shorey et al., 2012; Weng et al., 2012; Zhu et al., 2012). Our results provide new evidence that DIM induces apoptosis in HCC cells, consistent with the antitumor effects of DIM in other cancers. Apoptosis is associated with activation of caspases (Kim, 2016) and MAPK (White and Sacks, 2010). Cleaved-caspase3, which is critical in executing cell death (Maddika et al., 2007), was significantly elevated after exposure to DIM.

Several studies have shown that DIM has antitumor effects *via* the Hippo signaling pathway in gastric cancer cells (Li et al., 2013), ERK signaling pathway, as well as the MAPK and PI3K pathways in cervical cancer cells (Zhu et al., 2012). Nevertheless, the mechanisms underlying DIM-induced proliferation inhibition and apoptosis induction in HCC have yet to be fully clarified.

p38 MAPK has also been shown to produce anti-apoptotic effects (Nakagawa et al., 2004). DIM suppressed p38 MAPK in human retinal pigment epithelial cells (Park et al., 2015). Thus, p38 MAPKs might play a dual role: namely in mediating cell survival or promoting cell death through differential mechanisms (Grossi et al., 2014). Here we examined whether the p38 MAPK signaling pathway is functionally involved in the proliferation inhibition and apoptosis induction by DIM. We found that DIM was effective in inducing proliferation inhibition in hepatoma cells through activation of the p38 MAPK signaling pathway.

We investigated the molecular mechanism underlying the effect of DIM in suppressing tumor cell proliferation. Phosphorylation of p38 MAPK has been implicated in cellular responses to apoptosis (Zarubin and Han, 2005). The human MAPK pathway has been repeatedly shown to be down-regulated in cervical cancer cells (Meira et al., 2009) and considered as a potential therapeutic target in melanoma (Inamdar et al., 2010). Thus, we speculated that increased p38 phosphorylation might offer efficacy in DIM cancer therapy by inhibition of proliferation and induction of apoptosis. Lu et al. have previously demonstrated that inhibition of p38 prevented p38-mediated apoptosis in HCC cells (Lu et al., 2015), showing that the selective p38 MAPK inhibitor SB203580 attenuated the DIM-induced apoptosis. Taken together, these studies indicate that p38 activation is critical in the induction of apoptosis by DIM.

p38 MAPK signaling has been shown to promote tumor growth (Voisset et al., 2013), and enhanced p38 MAPK phosphorylation has been correlated with poor overall survival in patients with HER-2 negative breast cancer (Esteva et al., 2004) or with HCC (Wang et al., 2012). The induction of apoptosis by an improved triptolide derivative is closely associated with down-regulation the PCNA, a proliferation marker (Wang et al., 2017). In this study, PCNA was found to be down-regulated in response to DIM treatment along with reduced proliferation and enhanced phosphorylation of p38 MAPK in HCC cells, suggesting that the p38 MAPK pathway was involved in the growth inhibition of HCC cells by DIM. This is consistent with earlier reports using other tumor cell types (Khwaja et al., 2009; Vivar et al., 2009). Furthermore, the DIM-induced proliferation inhibition in HCC cells was attenuated by treatment with a pharmacological inhibitor of p38 MAPK.

Prolonged cytosolic elevation of Ca^2+^ might be toxic and trigger cell death. Studies have shown that DIM-induced interferon-γ expression in MCF-7 human breast cancer is mediated by p38, which is dependent upon cytosolic calcium signaling (Xue et al., 2005). Lim et al. found that induction of apoptosis by carvacrol, a monoterpenoid phenol, is mediated by depolarization of overloaded mitochondrial calcium (Lim et al., 2019). We observed an increase in [Ca^2+^]_i_ in response to DIM, which suggests DIM may induce sensitivity in HCC by causing disruption of cytosolic Ca^2+^ homeostasis.

We further noted that BAPTA-AM effectively prevented an increase in DIM-induced [Ca^2+^]_i_ and that DIM-induced apoptosis could be partially blocked by BAPTA-AM. Cheng et al. found that elevated cytosolic calcium concentration is inseparable from apoptosis in hepatoma cells (Cheng et al., 2011). Therefore, we posited that a continuously increasing cytosolic Ca^2+^ level might disturb the fine-tuned Ca^2+^ homeostasis and trigger a cascade of events resulting in tumor cell apoptosis. Arachidonic acid-induced calcium release from the endoplasmic reticulum is a critical step of the signaling pathway leading to JNK activation in bone marrow stromal cells (Rizzo et al., 1999a; Rizzo et al., 1999b). Therefore, we hypothesized that cytosolic calcium might be involved in the upstream signaling events leading to DIM-induced p38 MAPK activation and subsequent apoptosis. Pre-treatment with BAPTA-AM followed by DIM was shown to decrease phosphorylated p38 levels, indicating that cytosolic calcium elevation mediated the DIM-induced activation of p38. The activated p38 subsequently led to SMMC-7721 and HepG2 cell apoptosis. Although elevation of [Ca^2+^]_i_ may not be the primary cause for DIM cytotoxicity in HCC cells, since chelating free calcium with BAPTA-AM did not alter DIM-induced effects on cell proliferation, we found that [Ca^2+^]_i_ chelation attenuated DIM-induced apoptosis. Taken together, these observations suggest that DIM requires Ca^2+^ signaling to activate p38 MAPK and subsequent apoptosis.

A23187 is a selective Ca^2+^ ionophore and is commonly used to increase [Ca^2+^]_i_ in intact cells (White and Sacks, 2010). Increased intracellular calcium concentration by calcium ionophore A23187 aggravated the tamoxifen induced-proliferation inhibition in skin squamous cell carcinoma cells (Hasegawa et al., 2018). In the present study, co-treatment with DIM and A23187 at non-cytotoxic levels enhanced the proliferation inhibition of DIM. Co-treatment with A23187 and DIM in liver cancer cells also enhanced cell apoptosis in both cell lines, suggesting the calcium ionophore could enhance the effects of DIM. Modulation of Ca^2+^ influx has been previously shown to regulate several processes relevant to cancer, including cellular proliferation (Azimi et al., 2014). The A23187-treated C2C12 myoblasts cells occur in a manner distinct from its common effect on mitochondrial functions (Chmielewska and Malinska, 2011). A23187 administration led to a transient increase in cytosolic calcium levels, concomitant activation of calpain and a decrease in state 3 respiration rates, indicating mitochondrial dysfunction in C2C12 myoblasts, too. A23187 influenced of the opener on intracellular calcium levels led to elevation of [Ca^2+^]_i_ (Malinska et al., 2010), A23187 had effects on mitochondrial respiration and ATP production, emphasizing the multiple mechanisms of A23187 to enhance the anti-cancer effect of DIM, and the effects of this molecule on mitochondrial respiration and ATP production deserves further exploration in HCC. Overall, enhancement of cytosolic calcium may offer a novel therapeutic route for chemotherapy in the treatment of liver cancer.

## Conclusion

Cytosolic free calcium-dependent activation of p38 MAPK plays a key role in the anti-cancer effects of DIM. Combination of DIM with Ca^2+^ ionophore may serve as a new therapeutic approach to enhance chemotherapy efficacy in HCC.

## Data Availability Statement

The datasets generated for this study are available on request to the corresponding author.

## Author Contributions

RL, JC, YY, and BW conceived the study. YJ, YF, and XX performed the experiments, analyzed the data, and wrote the manuscript draft. MA and JG organized the data, helped with data interpretation, and reviewed the final manuscript.

## Conflict of Interest

The authors declare that the research was conducted in the absence of any commercial or financial relationships that could be construed as a potential conflict of interest.
